# Lobar graft evaluation in cadaveric lobar lung redo transplantation after living-donor lobar lung transplantation: a case report

**DOI:** 10.1186/s40792-024-02046-x

**Published:** 2024-10-23

**Authors:** Yui Watanabe, Tatsuaki Watanabe, Takashi Hirama, Sho Murai, Kazunori Ueda, Hisashi Oishi, Miki Akiba, Toshikazu Watanabe, Takaya Suzuki, Hirotsugu Notsuda, Ken Onodera, Takeo Togo, Hiromichi Niikawa, Masafumi Noda, Yoshinori Okada

**Affiliations:** 1 Department of Thoracic Surgery, Institute of Development, Aging and Cancer, Tohoku University, 4-1 Seiryomachi, Aobaku, Sendai, Miyagi 980-8575 Japan; 2 Organ Transplant Center, Tohoku University Hospital, Sendai, Japan

**Keywords:** Lung transplantation, Lobar lung transplantation, Donor assessment, Lung ultrasound

## Abstract

**Background:**

Lung transplantation is a vital option for patients with end-stage lung disease. However, it faces a significant challenge due to the shortage of compatible donors, which particularly affects individuals with small chest cavities and pediatric patients. The novel approach of cadaveric lobar lung transplantation is a promising solution to alleviate the donor shortage crisis. Both the mid-term and long-term outcomes of lobar lung transplantation are comparable to those of standard lung transplantation. However, patients undergoing lobar lung transplantation reported a significantly higher rate of primary graft dysfunction compared to patients undergoing standard lung transplantation. Therefore, careful donor selection is critical to improve outcomes after lobar transplantation. However, no established method exists to evaluate each lung lobar graft of deceased donors. This case report describes a case of cadaveric lobar lung transplantation to overcome size mismatch and donor shortage, with particular emphasis on lobar graft evaluation.

**Case presentation:**

A 39-year-old woman with scleroderma-related respiratory failure was listed for deceased donor lung transplantation due to a rapidly progressing disease. Faced with a long waiting list and impending mortality, she underwent bilateral living-donor lobar lung transplantation donated by her relatives. Post-transplant complications included progressive pulmonary vein obstruction and pleural effusion, which ultimately required retransplantation. An oversized donor with pneumonia in the bilateral lower lobes was allocated. Lung ultrasound was used to evaluate each lung lobar graft during procurement. The right upper and middle lobes and left upper lobe were confirmed to be transplantable, and lobar lung redo transplantation was performed. The patient’s post-transplant course was uneventful, and she was discharged home and returned to her daily activities.

**Conclusions:**

This case highlights the clinical impact of cadaveric lobar lung transplantation as a feasible and effective strategy to overcome the shortage of donor lungs, especially in patients with small thoracic cavities. By establishing donor lung evaluation techniques and overcoming anatomical and logistical challenges, cadaveric lobar lung transplantation can significantly expand the donor pool and offer hope to those previously considered ineligible for transplantation.

## Background

Lung transplantation (LTx) is an established treatment for end-stage lung disease. However, the growing waiting list highlights a shortage of suitable donors, especially for adults with small chest cavities and pediatric patients [1, 2]. Pediatric candidates, particularly those under 12, face higher mortality and longer wait times [1, 2]. Developed in the early 1990s, living-donor lobar lung transplantation (LDLLT) provides smaller grafts for urgent cases [3] but has declined with the advent of urgency-based deceased donor systems [4], remaining crucial only in countries like Japan with persistent organ shortages [5].

A significant contributor to the shortage of donor lungs is the high percentage of lungs that are considered unsuitable for transplantation, often due to complications such as edema following brain death or pneumonia from extended ventilator use in intensive care units (ICU). Nevertheless, some lung lobes may remain functional despite damage elsewhere, making them potential candidates for transplantation. Given the scarcity of transplantable lungs, cadaveric lobar lung transplantation (LLTx) has emerged as a viable alternative to address the shortfall of appropriately sized donor lungs [6, 7]. Recent research indicates that the mid- and long-term outcomes of LLTx are comparable to those of traditional LTx [8]. However, LLTx recipients often face a higher risk of severe primary graft dysfunction and longer ICU and hospital stays than those receiving standard LTx [8]. Additionally, acute mortality rates are higher among LLTx recipients [8].

However, LLTx can be a lifesaving option for patients such as our presenting case, who have no other alternatives and are on a waiting list in desperate need. Even a disabled donor could be allocated to a lower-ranked waiting list, thereby increasing the chances of finding a suitable match for these patients.

## Case presentation

A 39-year-old woman with a history of scleroderma developed respiratory failure due to collagen-related interstitial pneumonia. She was referred to our hospital for LTx and was listed for deceased donor LTx. However, due to the rapid progression of her disease and her small stature (height 150 cm), it was considered difficult for her to remain on the waiting list. Therefore, she underwent bilateral LDLLT (pulmonary vein anastomoses were performed over-and-over suture using 5-0 polypropylene suture), with the right lower lobe donated by her brother and the left lower lobe donated by her husband.

**Fig. 1:**
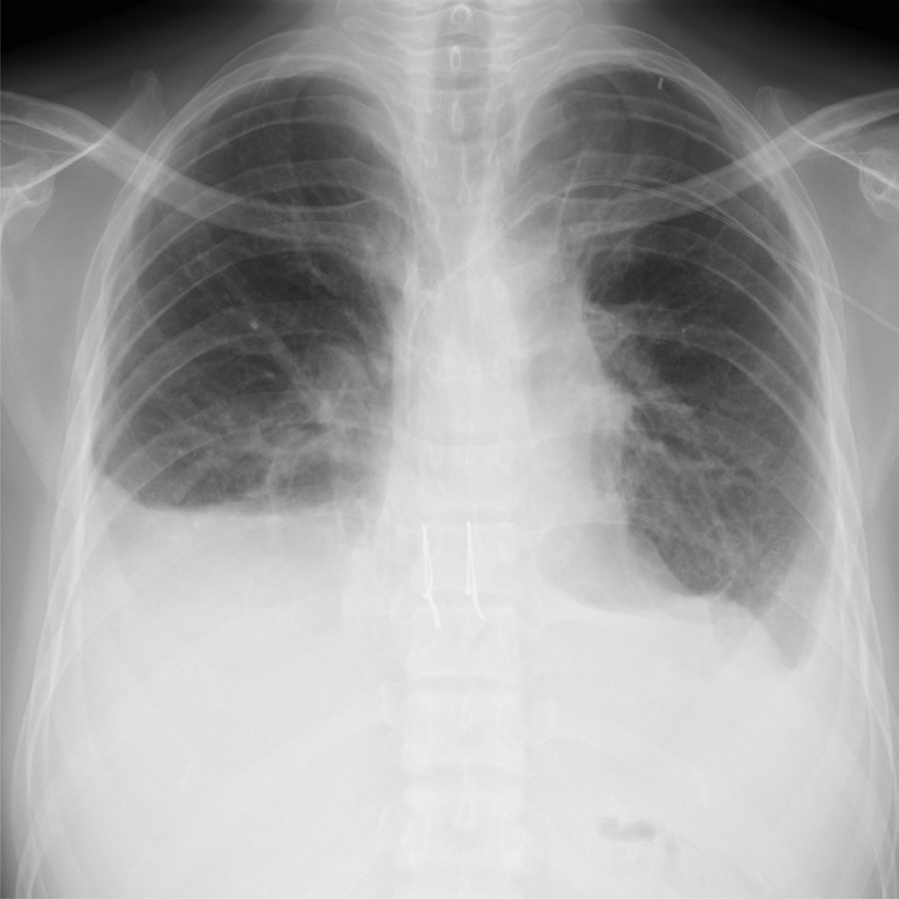
Chest radiograph before retransplantation showing pleural effusion in bilateral chest cavities

**Fig. 2:**
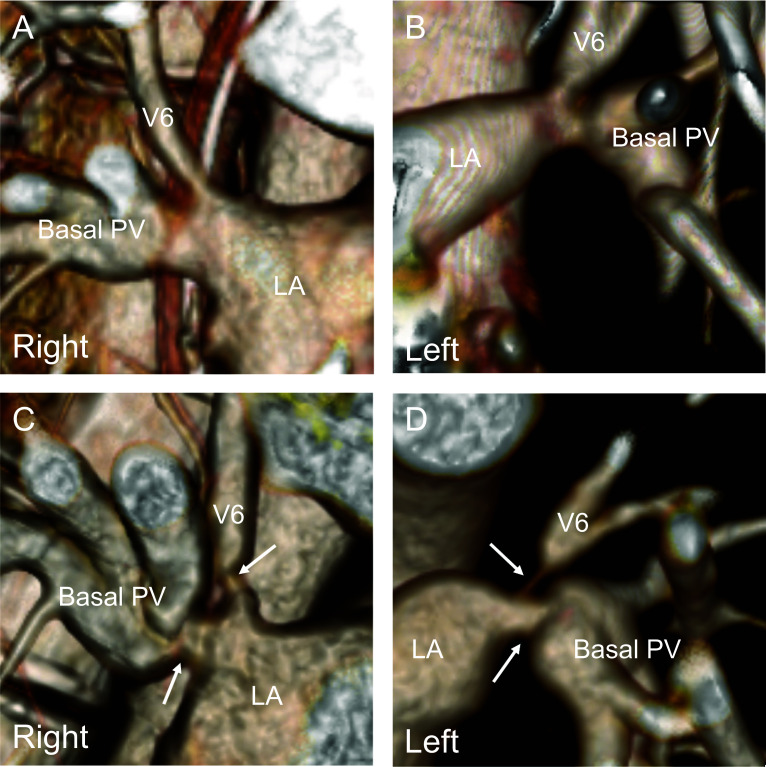
Three-dimensional computed tomographic angiography (**A** and **B** 5 days after living lobar lung transplantation, C and D 6 months after living lobar lung transplantation) showing progressive bilateral pulmonary vein obstructions (arrows). PV: pulmonary vein, LA: left atrium

The patient was discharged home 3 months after LDLLT and able to exercise. However, she was hospitalized in the sixth and seventh months after LDLLT for pleural effusion requiring diuretic treatment. In the eighth month after LDLLT, she was readmitted to the hospital for pleural effusion and pulmonary edema, as seen on a chest X-ray (Fig. 1), requiring ICU care. A thorough examination revealed progressive bilateral pulmonary vein stenoses (Fig. 2), which was regarded as a complication known as pulmonary vein obstruction (PVO) caused by intimal hyperplasia from the anastomotic site toward upstream of the veins [9, 10]. Therefore, it was difficult to save her life except by retransplantation, and she was placed on the deceased donor LTx waiting list 14 months after her LDLLT.

The following donor occurred on the 82nd day after the patient was listed. The donor was an allogeneic male in his 50 s. In the donor's preoperative evaluation, the ratio of partial pressure arterial oxygen and a fraction of inspired oxygen (P/F) was 310. However, chest radiographs showed increased infiltrative shadows on the mediastinal side from the right lung hilum to the middle and lower lung fields and the left middle and lower lung fields (Fig. 2A). Chest computer tomography (CT) also showed atelectasis and infiltrative shadows in the bilateral lower lobes. Bronchoscopy showed a large amount of white viscous sputum in the central airways, sputum accumulation in almost all bronchi on the right side, and mucosal edema and upwelling of sputum in the right lower lobe bronchus. The culture of respiratory secretions was positive for Staphylococcus aureus. The predicted vital capacity of the donor lungs calculated using the Japanese Respiratory Society equation [11] was 4196 mL, + 43.7% oversized graft for our patient, and she was initially not a high priority for transplantation. However, all lung transplant programs except our institution declined the donor. Therefore, the donor was allocated to our patient as the 316th candidate with both lungs available for bilateral LTx. Due to the + 43.7% oversized graft and bilateral lower lobe pneumonia, a lobar lung transplantation was considered.

**Fig. 3:**
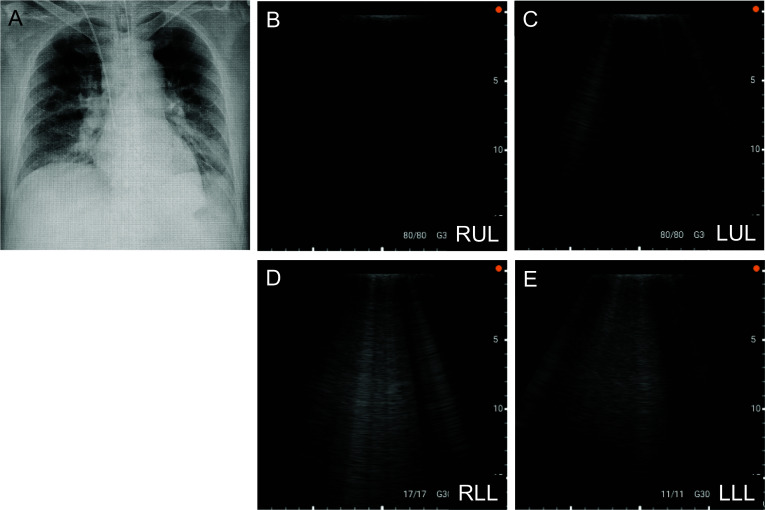
Chest radiographs showing increased infiltrative shadows on the mediastinal side from the right lung hilum to the middle and lower lung fields and the left middle and lower lung fields (**A**). Lung ultrasound showing that RUL (**B**) and LUL (**C**) were in the hypoechoic area. In contrast, RLL (**D**) and LLL (**E**) show numerous B-lines, indicating thickening of the interlobular septal wall and fluid retention in the alveoli. RUL: right upper lobe, RLL: right lower lobe, LUL: left upper lobe, LLL: left lower lobe

During the intraoperative lobar evaluation, the right upper and middle lobes and left upper lobe appeared transplantable based on subjective assessment such as visual inspection, palpation, and deflation test. Lung ultrasound using a portable device (Miruco, SIGMAX, Tokyo, Japan) showed that the right upper and middle lobes and left upper lobe were in the hypoechoic area (Fig. 3B and C). In contrast, the bilateral lower lobes showed numerous B-lines, indicating thickening of the interlobular septal wall and fluid retention in the alveoli (Fig. 3D and E). Blood gas analysis by direct puncture of the donor pulmonary vein (PV) showed a left superior PV of 534 mmHg, a right superior PV of 428 mmHg, and a right inferior PV of 75.3 mmHg. The left inferior PV was not sampled due to unstable circulation. The right upper and middle lobes and left upper lobe were well expanded, but the bilateral lower lobes were heavy and collapsed.

**Fig. 4:**
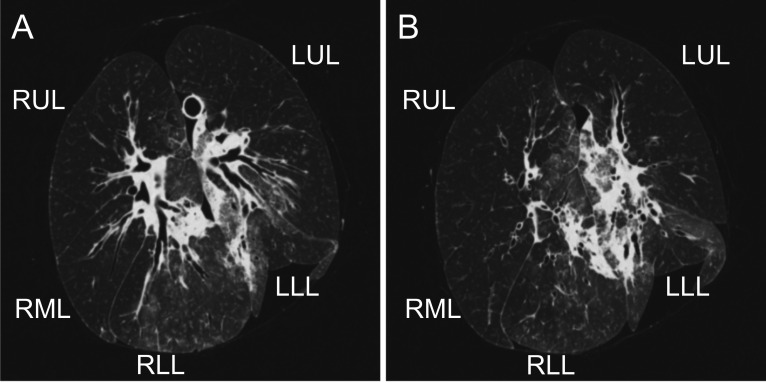
Ex vivo lung CT showing severe infiltration and consolidation in RLL and LLL (**A** and **B**). RUL: right upper lobe, RML: right middle lobe, RLL: right lower lobe, LUL: left upper lobe, LLL: left lower lobe

After transportation to our hospital, ex vivo lung CT, which is performed in all our cases from deceased donors [12], showed severe infiltration and consolidation in the bilateral lower lobes (Fig. 4A and B). Redo transplantation was performed under cardiopulmonary bypass. Both lower lobes were resected on the back table, and the left upper lobe was transplanted first as the left lung. Left-side anastomoses are as follows: recipient’s left main bronchus to donor’s left upper bronchus, recipient’s left main pulmonary artery (PA) to donor’s left main PA, and recipient’s left atrium (LA) to donor’s LA (LA anastomoses were performed everting mattress running suture using 4–0 polypropylene suture). The right upper and middle lobes were transplanted as the right lung. Right side anastomoses are as follows: recipient’s right main bronchus to donor’s right main bronchus, recipient’s right main PA to donor’s right main PA, and recipient’s LA to donor’s LA. The donor’s right lower bronchial stump was covered with a polyglycolic acid sheet. The ischemia time was 6 h 52 min for the left lung and 9 h 33 min for the right lung. The operation time was 15 h and 32 min, and the blood loss was 6063 g.

**Fig. 5:**
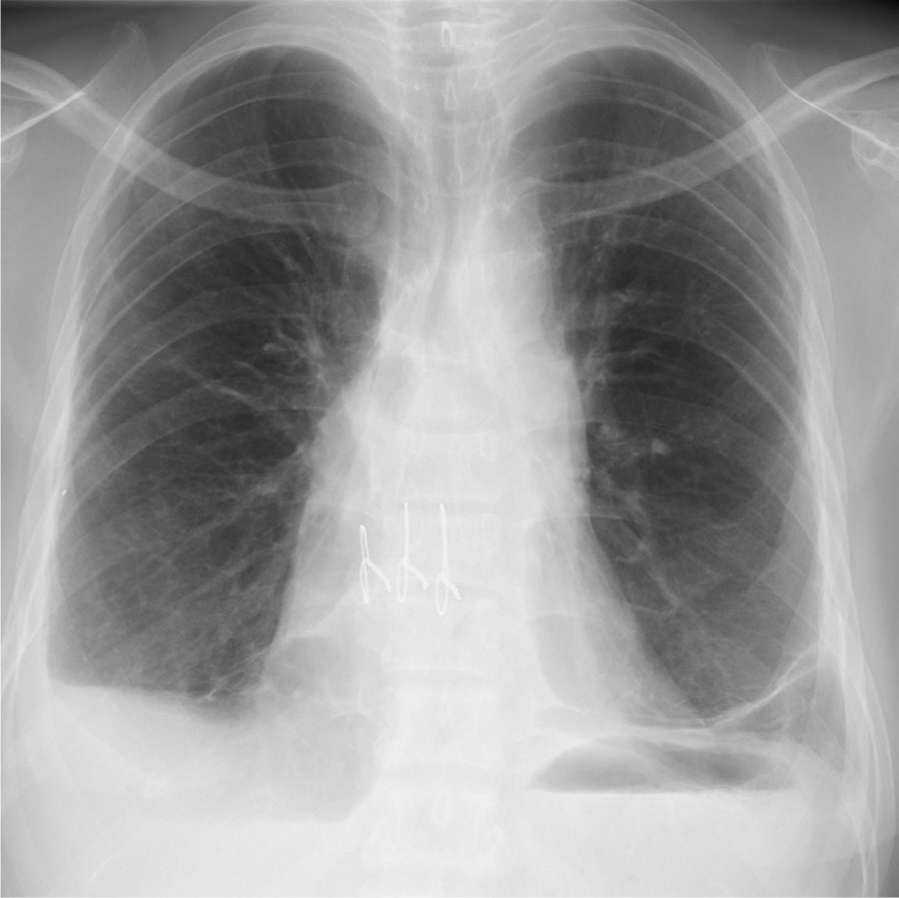
Chest radiograph just before discharge showing fully expanded clear lung fields

The patient’s post-transplantation course was good, and the International Society for Heart and Lung Transplantation primary graft dysfunction score was three at 24 h, two at 48 h, and 0 at 72 h, which improved with time. The patient was weaned from the ventilator on post-transplant day 14 and left the intensive care unit on post-transplant day 16. She was discharged home on day 58 after transplantation (Fig. 5). Her measured lung capacity nine months after transplantation was 1990 mL, and her general condition is also now good. Current chest CT showed fully expanded bilateral transplanted lungs and no dead spaces. There are no issues in the bronchial stump.

## Discussion

LLTx has been reported to have a high acute mortality rate [8], so graft selection is essential. However, there is no established graft evaluation method for each lobar graft. We established a large animal model of the left upper LLTx [13]. We introduced lower lobe aspiration pneumonia and acute lung injury in this donor to investigate the evaluation method of the upper lobar graft [14]. The results showed that lung ultrasound assessment was most correlated to post-transplant outcomes rather than visual inspection, palpation, and differential pulmonary vein blood gas analysis, and we applied it clinically. Although direct PV puncture for blood gas analysis is valuable, it is only sometimes practical to expose four pulmonary veins, draw blood, and wait for the results during procurement, when decisions must be made quickly. Lung ultrasound, on the other hand, is quick and easy to perform. Our experiments using a large animal model showed that the proportion of B-lines, the abnormal comet sign indicating increased lung density due to pulmonary edema on lung ultrasound, of the grafts with P/F of more than 300 after transplantation were less than 50% [14]. These findings from the large animal study need to be validated in future clinical cases.

In this case, LDLLT was initially performed. PVO occurs after PV anastomosis with the progression of intimal hyperplasia from the anastomotic site to the upstream PVs [9, 10]. Vascular complications following lung transplantation are observed in 1–3% of patients and are linked to graft failure, the need for retransplantation, and a high mortality rate [10]. Typically, these complications involve issues with the pulmonary arteries or pulmonary vein thrombosis. PVO is uncommon, and its frequency of occurrence remains uncertain. In LDLLT, keeping the adequate length of the donor inferior PV in living-donor lobectomy is important for PV anastomosis of the recipient. In this case, the donor inferior PVs in the bilateral lobar grafts were not long, and the suture lines of PV anastomoses were placed almost at the bifurcation of the V6 and the basal PV, respectively, which may have played a role in the development of clinically significant PVO. PVO is caused by suture irritation and by holding the wall during anastomosis, which irritates the endothelium [9, 15]. Thus, maintaining the appropriate length of the donor inferior PV in living-donor lobectomy and minimizing contact with the vessel wall may be crucial in preventing this complication.

In Japan, organ donation for LTx requires an ABO-identical or compatible, and an ABO-identical is given priority. When the donor is under 18, preference is given to a recipient under 18. If both donor and recipient are over 18, preference is given to grafts within 30% of graft size. While pediatric patients on the waiting list have the opportunity to receive a donation from a pediatric donor, small female patients on the waiting list often have fewer transplant opportunities and a more extended waiting period. In this case, all identical blood group (type O) and compatible blood group (non-type O) candidates within 30% of the graft size had declined, so the patient (type O, + 43.7%) was placed as the 316th candidate, and both lungs were available for LLTx. LLTx can be a lifesaving procedure for small adult patients on a waiting list.

The bilateral lower lobes were considered ineligible for transplantation during donor evaluation. The left upper lobe was transplanted without a bronchial stump on the left side. On the right side, deciding whether to transplant only the upper lobe or the upper and middle lobes was complex. The anatomy of the right lung differs from that of the left lung, and the upper LLTX avoids the bronchial stump remnant, but the volume of the transplanted lung is smaller. In this case, the predicted lung volumes by the segment-counting method [16] after the right and left upper LLTx were 1767 mL (60.5%). In the upper and middle LLTx, the bronchial stump after the lower lobe resection remains in the transplanted lung. However, a larger lung volume was obtained, and the predicted lung volumes after transplantation of the right upper and middle lobes and left upper lobe were 2208 mL (75.6%), and her measured lung capacity nine months after transplantation was 1990 mL (66.5%).

There is much controversy regarding the treatment of the bronchial stump in LLTx. Some surgeons argue that the recipient’s right lower lobe bronchial stump is acceptable, but the donor lung's right lower lobe bronchial stump should be avoided [17]. In contrast, other surgeons believe the donor’s right lower lobe bronchial stump is acceptable [18]. In this case, the recipient’s bronchi were only up to the main bronchus, and the donor's right lower lobe bronchial stump could not be avoided to ensure sufficient lung volume including middle lobe. Therefore, although there was a particular risk of bronchial fistula, transplantation of the right upper and middle lobes and left upper lobe was decided, and no post-operative bronchial fistula was observed.

## Conclusions

In conclusion, LLTx is a crucial option for patients with limited alternatives, especially when long waitlists and high mortality risks make standard transplants unfeasible. Advanced methods like lung ultrasonography help identify usable lung portions for LLTx.

## Data Availability

Data will be made available on reasonable request.

## Abbreviations

CT: Computed tomography
ICU: Intensive care unit
LA: Left atrium
LDLLTx: Living-donor lobar lung transplantation
LLTx: Lobar lung transplantation
LTx: Lung transplantation
PA: Pulmonary artery
P/F: The ratio of partial pressure arterial oxygen and a fraction of inspired oxygen
PV: Pulmonary vein
PVO: Pulmonary vein obstruction
